# Infants show systematic rhythmic motor responses while listening to rhythmic speech

**DOI:** 10.3389/fpsyg.2024.1370007

**Published:** 2024-06-17

**Authors:** Natalie Boll-Avetisyan, Arina Shandala, Alan Langus

**Affiliations:** ^1^Department of Linguistics, University of Potsdam, Potsdam, Germany; ^2^Research Focus Cognitive Sciences, University of Potsdam, Potsdam, Germany; ^3^International Doctorate for Experimental Approaches to Language and Brain (IDEALAB), University of Groningen, Netherlands/University of Newcastle, United Kingdom/University of Potsdam, Germany and Macquarie University, Sydney, NSW, Australia

**Keywords:** infants, rhythm perception, rhythmic body movements, rhythmic cues, speech segmentation, individual differences

## Abstract

Rhythm is known to play an important role in infant language acquisition, but few infant language development studies have considered that rhythm is multimodal and shows strong connections between speech and the body. Based on the observation that infants sometimes show rhythmic motor responses when listening to auditory rhythms, the present study asked whether specific rhythm cues (pitch, intensity, or duration) would systematically increase infants’ spontaneous rhythmic body movement, and whether their rhythmic movements would be associated with their speech processing abilities. We used pre-existing experimental and video data of 148 German-learning 7.5- and 9.5-month-old infants tested on their use of rhythm as a cue for speech segmentation. The infants were familiarized with an artificial language featuring syllables alternating in pitch, intensity, duration, or none of these cues. Subsequently, they were tested on their recognition of bisyllables based on perceived rhythm. We annotated infants’ rhythmic movements in the videos, analyzed whether the rhythmic moving durations depended on the perceived rhythmic cue, and correlated them with the speech segmentation performance. The result was that infants’ motor engagement was highest when they heard a duration-based speech rhythm. Moreover, we found an association of the quantity of infants’ rhythmic motor responses and speech segmentation. However, contrary to the predictions, infants who exhibited fewer rhythmic movements showed a more mature performance in speech segmentation. In sum, the present study provides initial exploratory evidence that infants’ spontaneous rhythmic body movements while listening to rhythmic speech are systematic, and may be linked with their language processing. Moreover, the results highlight the need for considering infants’ spontaneous rhythmic body movements as a source of individual differences in infant auditory and speech perception.

## 1 Introduction

It is widely acknowledged that the perception of speech rhythm helps infants to tune into the language that surrounds them (Gleitman and Wanner, 1982; Langus et al., 2017). Newborns can already distinguish between languages that differ in their overall speech rhythm (Nazzi et al., 1998; for a meta-analysis, see Gasparini et al., 2021). At 4–6 months, infants have internalized their native languages’ metrical structure (Friederici et al., 2007; Höhle et al., 2009), and from 7 months onwards they can use rhythmic cues for identifying words (e.g., Echols et al., 1997; Jusczyk et al., 1999; Abboub et al., 2016) and phrases (Hirsh-Pasek et al., 1987; Nazzi et al., 2000) in continuous speech. These abilities are an important step for acquiring words and syntax (Gleitman and Wanner, 1982; Christophe et al., 1994; Jusczyk, 1997; Weissenborn and Höhle, 2001), and predict later language skills (Newman et al., 2006; Junge et al., 2012; Cristia et al., 2014; Höhle et al., 2014; Marimon et al., 2022). Surprisingly, only a few studies investigating the role of rhythm in language acquisition have considered that rhythm is multimodal and has intrinsic connections between speech and the body, although it is well established that regular rhythm – such as in music – facilitates sensorimotor synchronization of bodily movements to perceived rhythmic regularities. Hence, if infants perceive rhythm in spoken language, they might express this sensitivity by showing rhythmic engagement with the speech rhythm. The goal of the present study was thus to explore whether infants spontaneously produce systematic rhythmic body movements while listening to the rhythm of spoken language and whether such rhythmic engagement supports the perception and acquisition of spoken language.

Our investigation of the potential link between infants’ body and speech rhythm perception was inspired by a coincidental observation of infants’ spontaneous body movements in experiments that investigate their language development. Language acquisition research often employs artificial language learning paradigms for studying what type of speech cues infants rely on for extracting word-like units from continuous speech. In this paradigm, infants are familiarized with artificial miniature languages that are highly reduced nonsense speech streams. After listening to these streams for a few minutes, infants are tested on their recognition of specific syllable combinations that were or were not part of the artificial speech stream (for example, the co-occurrence probabilities of syllables: Saffran et al., 1996; Aslin et al., 1998; rhythmic/prosodic cues: Thiessen and Saffran, 2003; Abboub et al., 2016; Marimon et al., 2022; phonotactic patterns: Mintz et al., 2018). These artificial languages are often designed such that they present syllables organized in a repetitive order resulting in a highly rhythmic auditory signal that seems to facilitate the perception of these artificial languages (Johnson and Tyler, 2010; Lew-Williams and Saffran, 2012; Mersad and Nazzi, 2012; Marimon et al., 2022). Interestingly, when running such experiments, we have also observed that many infants spontaneously move their bodies rhythmically, as if dancing to the rhythm of these artificial languages. Here we therefore investigate whether such rhythmic motor engagement with the rhythmic speech in artificial language learning experiments reflects infants’ processing of the speech rhythms, and whether individual differences in motor engagement is associated with their speech processing performance.

There is some evidence that speech perception and bodily movements are intrinsically connected. Previous research into the multimodality of speech perception has drawn a link between sensorimotor information and speech perception by showing that phoneme perception is modulated by restricting lip movements in infants as early as 4.5 months of age (Yeung and Werker, 2013; Bruderer et al., 2015). Beyond articulatory gestures, when speaking, humans’ body gestures move in synchrony with speech prosody, that is, the melody and rhythm of speech (e.g., Wilson and Wilson, 2005; Schmidt-Kassow and Kotz, 2008; Cummins, 2009; Guellai et al., 2014). In fact, hand movements are typically entrained with the prosodically strongest syllables of words and phrases (e.g., De Ruiter, 1998). This synchrony between speech prosody and hand movements starts to emerge when infants produce their earliest babbles (Esteve-Gibert and Prieto, 2014). Also, when listening to speech, infants have been found to follow the rhythm of speech with their body movements from birth (e.g., Condon and Sander, 1974; Mundy-Castle, 1980; Papoušek et al., 1991; Papoušek, 1992; Masataka, 1993; Kuhl et al., 1997). While spoken language is not perfectly rhythmic, it can assume a regular rhythm in many everyday activities including music, poetry, and nursery rhymes, where the regular metrical rhythmic patterns occur in an exaggerated form. In the context of these highly regularized rhythms, the link between body and perception is even more noteworthy: across cultures, both adults and children dance, bounce, or tap in synchrony with the beat when listening to music (e.g., Brown et al., 2004; Kirschner and Tomasello, 2009; Fujii et al., 2014). This raises the question of whether the association of body rhythm and speech rhythm has a function in language perception and acquisition.

Young infants occasionally show rhythmic body movements that have been described as vegetative reflexes produced by the limbs, torso, and head (Thelen, 1981). While these movements have been suggested to be precursors of more coordinated rhythmic movements in dancing (Thelen, 1981), theories have also highlighted their role as a transitional point to later communication abilities in the first year of life (Iverson and Thelen, 1999). For example, rhythmic movements of the hands and the arms have been linked with the maturation of oral articulators, with an observed decrease in produced rhythmic body movements occurring around the time when infants start producing more vocalizations (Iverson and Thelen, 1999). Whereas very young infants move rhythmically even in absolute silence, these movements are enhanced in social, interactive contexts, when the caregiver enters a room, or when infants are being presented with a toy (Thelen, 1981). This suggests that the development of rhythmic movements may be linked to communicative processes, of which language forms part.

While Thelen (1979, 1981) studies were purely observational, controlled studies have attested that the degree to which infants spontaneously produce rhythmic motor movements seems to depend on specific auditory conditions. For example, Zentner and Eerola (2010) explored the rhythmic motor engagement of 5- to 24-month-old infants under experimentally controlled conditions in a laboratory when listening to different types of music (i.e., isochronous drumbeats and naturalistic music) and speech (i.e., naturalistic adult- and infant-directed French and English). Results revealed that all age groups engaged less rhythmically when listening to speech than when listening to music. Furthermore, the duration of infants’ movements in this study was faster to isochronous beats with faster tempi. These results from Finnish and Swiss infants were replicated with Brazilian infants in a study by Ilari (2015), which additionally showed that Brazilian infants tended to produce more movement to music stimuli than the European infants in Zentner and Eerola (2010). This may indicate that the development of spontaneous rhythmic motor responses to auditory signals is influenced by the culture that infants are surrounded by (Note that previous studies also more generally report cross-cultural differences regarding infants’ gross motor activities; e.g., Bril and Sabatier, 1986; Victora et al., 1990; Venetsanou and Kambas, 2010, so the question remains whether the cross-cultural differences depend on the auditory context in Zentner and Eerola’s and Ilari’s studies).

The understanding of infants’ spontaneous rhythmic movements to different auditory stimuli was further refined in a study by de l’Etoile et al. (2020), which focused on 6- to 10-month-old infants. Their results indicated that infants’ movements were more regular when they were listening to regular beats than when the beat was irregular. Moreover, a longitudinal study by Mazokopaki and Kugiumutzakis (2009) followed infants’ development of spontaneous rhythmic vocalizations and movements from 2 to 10 months of age. According to their results, infants produced more vocalizations, hand gestures and dance-like movements when hearing baby songs than when their behavior was observed in silence. However, Fujii and colleagues (Fujii et al., 2014), who explored 3- to 4-month-old infants, did not observe more limb movements when infants listened to pop music than when there was silence but found the presence of music to influence infants’ vocal quality. Empirical studies thus suggest that infants manifest an increase in their rhythmic movements to auditory rhythms, and that the quantity of movements is modulated by the type and the register of the auditory rhythms. The present study will extend this work by asking whether the quantity of rhythmic movements also depends on the type of speech rhythms.

Prior research on adult (e.g., Bolton, 1894; Woodrow, 1909, 1911; Hay and Diehl, 2007) and infant listeners (e.g., Yoshida et al., 2010) has established that different acoustic types of speech rhythms lead to differences in rhythmic perception: Streams of sounds alternating in duration (short-long-short-long…) tend to be perceived as iambic (i.e., binary parsings with a weak-strong stress pattern), but streams of sounds alternating in intensity (loud-soft-loud-soft…) or pitch (high-low-high-low…) tend to be perceived as trochaic (i.e., binary parsings with a strong-weak stress pattern). The finding that this rhythmic grouping asymmetry is consistent across speakers of many different languages (Hay and Diehl, 2007; Bhatara et al., 2016; Boll-Avetisyan et al., 2020b) has given rise to proposals that the rhythmic grouping biases are based on an innate domain-general auditory mechanism (Hayes, 1995; Nespor et al., 2008). However, in adults, the magnitude of the effect seems to depend on experience (e.g., language experience: Iversen et al., 2008; Bhatara et al., 2013, 2016; Crowhurst and Olivares, 2014; Langus et al., 2016; music experience: Boll-Avetisyan et al., 2016) and individual skills (i.e., musical aptitude: Boll-Avetisyan et al., 2017, 2020a), indicating individual variability in the reliance of this perceptual mechanism that warrants further investigation, in particular in infants, for whom such data is yet lacking.

Research that addressed the potential functions of this auditory mechanism established that these rhythmic biases influence infants’ segmentation of artificial languages into word-like units (Bion et al., 2011; Hay and Saffran, 2012; Abboub et al., 2016). Abboub et al. (2016) (which serves as the basis for the current one) focused on two questions: what acoustic cues infants aged 7.5 month-old used to rhythmically group speech, and how linguistic experience influenced this use. They employed a cross-linguistic comparison of German- and French-learning 7.5-month-old infants to assess whether such rhythmic biases influencing speech segmentation were language-general or language-specific. In their study, infants were first familiarized with artificial language streams composed of syllables that alternated either in pitch, duration, or intensity, while all other rhythmic cues were kept constant, or to a stream that showed no rhythmic alternation (control condition). Following this familiarization, infants were tested on their recognition of syllable pairs. As a result, irrespective of language background, infants showed recognition of syllable pairs if they were familiarized with pitch- or duration-varied streams, but not when they heard intensity-varied or unvaried streams. They concluded that this result may speak for language-general rhythmic biases in young infants, noting that specific cues (here: duration, pitch) might be more accessible than others (here: intensity) for them. The present study aimed to build on Abboub et al. (2016) by investigating infants’ spontaneous rhythmic movements and whether the infants’ speech segmentation performance in that task was linked to their rhythmic movements.

Past research has revealed that infants’ speech segmentation skills are subject to individual variability. Factors that are associated with their speech segmentation performance are, for example, their babbling skills (Hoareau et al., 2019) and their later lexicon size (Newman et al., 2006; Junge et al., 2012), but also environmental factors such as the quantity of infant-directed speech input (Hoareau et al., 2019) and social interactions with their mother (Vanoncini et al., 2022, 2024). In segmentation experiments using the head-turn paradigm, individual differences are often revealed as follows. Infants first hear a speech stream, and in a subsequent test phase, it is probed whether they look longer to an unrelated visual stimulus (a lamp) while hearing familiar or unfamiliar test words. Generally, the direction of their looking preference, that is, whether they look longer when hearing the familiar or the unfamiliar/novel, is deemed irrelevant, as both directions indicate that infants at the group level perceived the difference between the stimuli. However, it has been noted (see Hunter and Ames, 1988 for a model) that infants who are more mature in their development (e.g., older infants) are more likely to express novelty preferences (i.e., they listen longer while hearing unfamiliar test words). Results of studies targeted at identifying predictors of infant speech segmentation performance are in line with this: novelty preferences are exhibited by infants who have more advanced babbling skills (Hoareau et al., 2019) and higher later word knowledge (Singh et al., 2012), reflecting that matureness in language development is associated with novelty preferences. Moreover, novelty preferences are more likely in infants whose mothers show less predictable social gaze behavior (Vanoncini et al., 2024) and whose mothers are more in emotional synchrony with their babies, suggesting that social aspects may also be a source of individual differences. The significant correlations between infants’ listening preferences and the probed predictors in these studies also signalize that the strength of infants’ preferences, expressed by the magnitude of their listening preferences, is increased in infants with a more mature language development. Following up on this background, we explored infants’ rhythmic body movements as another potential source of individual variability in infants’ speech segmentation performance.

With the present study, we addressed the question of whether rhythmic motor responses could have a function in language acquisition, following our assumption that infants’ multisensory experience of producing (motor) rhythm while perceiving (auditory) rhythm should reinforce the perception of the rhythmic structure of language. We asked two specific research questions: (1) whether infants would show systematic rhythmic movements in the presence of auditory rhythmic cues that guide their speech segmentation and (2) whether infants showing more rhythmic motor engagement would show a more mature (i.e., stronger) speech segmentation performance. To address this, we used video data from Abboub et al. (2016), which included recordings of 7.5-month-olds, and their unpublished data from 9.5-month-olds. We predicted that infants would show rhythmic body movements to the regular occurrence of the rhythmic pattern in the auditory stimuli in these experiments. We also predicted that infants would show more rhythmic movements when perceiving rhythms cued by pitch and duration (the two conditions prior studies found infants to be sensitive to, showing rhythmic grouping) than when cued by intensity or no prosodic property. Given that our data comprised two age groups (7.5 vs. 9.5 month), and that infants had been exposed to stimuli that were either pronounced as French or German, we additionally asked whether rhythmic movements would depend on these factors, but we did not have any specific predictions with regards to these. Regarding (2), we expected a positive relationship between infants’ speech segmentation performance and their quantity of rhythmic body movements, with more rhythmic movements being linked to a more mature speech segmentation performance (matureness expressed by novelty listening preferences and the magnitude of their listening preferences).

## 2 Materials and methods

### 2.1 Participants

For the present study, we used Abboub et al. (2016) data of German-learning 7.5-month-olds (*n* = 72; 38 female, mean age: 7.43 months, range: 7.00–8.30; the set originally included *n* = 80, but no videos were available for 8 infants) and added unpublished data of 9.5-month-olds (*n* = 76; 44 female, mean age: 9.47 months, range: 9.03–10.00; no videos were available for 4 additional babies). Table 1 indicates the infants’ distribution across conditions. Both of the samples did not include additional infants that were tested but removed due to Abboub et al.’s drop-out criteria. All infants were born full-term, had no reported family risk for language-related developmental disorders, and were growing up in monolingual households. Before the experiment, parents signed informed consent and filled out a demographic questionnaire. Ethics approval was obtained from the ethics committee at University of Potsdam.

**Table 1:** Overview of the number of participants per age and condition.

Condition	N of 7.5 months	N of 9.5 months
Pitch	20	18
Duration	20	18
Intensity	12	20
Control	20	20
Sum	**72**	76

### 2.2 Stimuli

In Abboub et al. (2016) original study, there were both familiarization and test stimuli. As familiarization stimuli, artificial language streams were used. These were rhythmic speech streams of six syllables consisting of six different vowels and consonants, which were concatenated into syllable streams with a 100 ms pause between each syllable. The speech stimuli were synthesized with the MBROLA software (Dutoit et al., 1996) with both a French (FR4) and a German pronunciation (DE5). The six syllables within a stream always co-occurred in the same fixed order (i.e., /na: zu: gi: pe: fy: ro: na: zu: …/), yielding a transitional probability of 1.0. This fixed order was repeated 66 times, to yield a stream that would last for 3 min. There were four conditions for marking prominence in the syllable streams: in the first three conditions, every second syllable in a stream was stressed (i.e., strong) by pitch, duration, or intensity cues. In the control condition, no syllable was stressed. Syllable durations, pitch and intensity values and ranges were similar to that of previous studies (e.g., Hay and Diehl, 2007; Bion et al., 2011; Bhatara et al., 2013), which were based on acoustic measurements of child-directed speech (for acoustic values, see Table 2). As test stimuli, six syllable pairs were used. Importantly, all test stimuli were flat in prosody, so that infants’ recognition of a syllable pair as familiar or novel could only be based on their identification of the phonemic/syllabic information of the test stimulus, and not on its rhythm. Three of these syllable pairs would have occured as strong-weak in the familiarized artificial language, and three that occur as weak-strong; that is, if an infant was familiarized with a stream alternating as /NA zu GI pe FY ro…/, three “strong-weak” test stimuli were /na zu/, /gi pe/ and /fy ro/, while the “weak-strong” test stimuli were /zu gi/, /pe fy/ and /ro na/. For more information about the choice of phonemes for the generation of syllables and a more detailed description of the stimuli, consult Abboub et al. (2016).

**Table 2:** Acoustic values of the rhythmic cues per condition.

	Condition	Pitch (Hz)	Intensity (dB)	Duration (ms)
Familiarization	Duration	200	70	260–460
	Intensity	200	66–74	360
	Pitch	200–420	70	360
	Control	200	70	360
Test	(All Conditions)	200	70	360

### 2.3 Procedure

In the original study, infants were tested using the head-turn preference procedure (HPP, Kemler Nelson et al., 1995). During the experiment, infants were seated on their caregiver’s lap in a soundproof booth. In front of the infant, there was a green light. On both sides of the room, there was a red light located on the same level as the green light. Loudspeakers were hidden under the red lights. Throughout the experiment, the caregiver was wearing headphones that played music to mask the stimuli the infant perceived. Video recordings of the experiment were made to verify the coding of infants’ looking times. During the experiment, stimulus presentation was controlled by the experimenter via blinking lights, which, depending on the infant’s head movement, were manipulated via button pressing. At the start of the experiment, infant’s gaze was centered with the green blinking light. Then, the experimenter would make one of the red lights blink to attract infant’s attention to it. When the infant looked at the blinking red light, the auditory stimulus would start.

During familiarization, infants listened to an artificial language stream with one of the four acoustic manipulations (pitch/duration/intensity/control). The sound came from both loudspeakers and lasted for 3 minutes. The blinking of the red light would stop if the infant turned away for more than 2 s, and in this case, the green light would begin blinking again (but the sound was never stopped during this phase). In the following test phase, which was the same for all infants regardless of familiarization conditions, segmentation was tested with two test trial types: “strong-weak” trials versus “weak-strong” trials. In sum, there were 12 test trials, half of which were “strong-weak” trials, and half of which were “weak-strong” trials. Test trials were constructed on the basis of the 6 syllable pair test stimuli (see 2.2), each containing 16 repetitions of one test stimulus (e.g., nazu nazu nazu). In order to arrive at 12 test trials, each of the 6 test trials was presented twice; once in trial position 1–6 (block 1), and once in trial position 7–12 (block 2). The order of test trials within a block was randomized across infants. Whether the stimuli were German- or French-sounding was counterbalanced (in our data: French-sounding: *n* = 78, German-sounding: *n* = 70). The test trials came randomly from either the right or the left side, in association with the blinking lamp on the same side. The blinking and the stimulus would stop if the infant turned away for more than 2 s, and in this case, the green light would begin blinking. All stimuli were played at comfortable volume (*ca.* 65 dB). In total, the experiment maximally took 6 min. Summed looking times per test trial (i.e., looking times per trial excluding the intervals during which the infant turned the head away from the side lamp) were taken to indicate infants’ interest in a trial. Following the standard HPP procedure, at the group level, the difference in looking time between the average of the three “strong-weak” and the average of the three “weak-strong” test trials can be taken to indicate that infants have recognized the difference between the test trial types, namely that one test trial type was familiar to them (i.e., the syllable pairs following from their grouping of the continuous artificial language stream, e.g., /na zu/ when familiarized with a /NA zu GI pe …/ stream, if they perceived a strong-weak grouping), and half of them was novel to them (i.e., the syllable pairs that would not follow from their perceived grouping; in this case, e.g., /zu gi/).

### 2.4 Data preprocessing and analysis

Video recordings were annotated for infants’ rhythmic body movements. Manual annotations were performed by three independent coders using the free annotation software ELAN (2016). For each video, coders identified and marked all intervals of rhythmic movements during the 3-min familiarization phase. Rhythmic movements were defined as comprising a minimum of three immediate repetitions of a bodily movement (Thelen, 1979) of any body part (e.g., limb, hand, legs). To test the reliability of rhythmic movement locations, an independent tester performed the inter-rater reliability check by calculating Cohen’s Kappa for a random subset of approximately 7% of the cases (11 cases, including six 7.5- and five 9.5-month-olds). The kappa value ranged from 0.72 to 0.86 for two different rater pairs, suggesting moderate to strong agreement (McHugh, 2012).

To determine the predictors of rhythmic movements, an analysis of variance (ANOVA) was run. As a dependent variable, we used infants’ total rhythmic moving times defined as the sum of all rhythmic intervals in milliseconds. Condition (pitch, intensity, duration or control), Age (7.5 or 9.5 months old) and Pronunciation (French or German-sounding) were between-participant fixed factors. Results are reported in section 3.1.

To investigate whether infants’ rhythmic motor engagement correlated with their performance in the speech segmentation task, we analyzed a subset of infants that exhibited rhythmic movements during the experiment (*n* = 62). We performed two correlation analyses, both of which used infants’ total rhythmic moving times (i.e., the sum of all intervals infants produced rhythmic movements while listening to the artificial language) as one variable, and both of which based the second variable on infants’ summed looking time per trial, with the following difference: For the *first* correlation analysis, we generated a difference (Δ) score of the infants’ looking times at test by subtracting the average of the looking times to “strong-weak” test trials from the average of the looking times to “weak-strong” test trials. Hence, positive Δ score values reflect longer looking times for “weak-strong” test trials, and negative Δ score values reflect longer looking times for “strong-weak” test trials. Similar difference scores were also used in Jusczyk and Aslin (1995), Singh et al. (2012), and Hoareau et al. (2019).

The *second* correlation analysis was motivated as follows: Since in the original study, the expected grouping depended on the acoustic cue (i.e., strong-weak grouping with pitch and intensity; weak-strong grouping with duration), pooled looking-time data may be difficult to interpret. However, because of the small sample size, it was impossible to further sub-set the data by the four conditions. Hence, we did the following: for the second correlational analysis, we changed the looking time Δ scores between trial types to *absolute* values by removing the positive/negative sign: all Δ scores were turned into positive values. Consequently, these absolute looking-time scores would reflect the magnitude of a preference, with higher values indicating more mature (i.e., stronger) listening preferences, independent of the direction of the preference. In both cases, this Δ score was based on looking times during the first six trials, following Abboub et al. (2016), who found that differences in listening preferences at test only occurred during the first of the two blocks in their study. The corresponding results are reported in section 3.2.

All statistical analyses and visualizations were done in R (version 4.3.2., R Core Team, 2021). The ANOVAs were performed using the package ez (version 4.4–0, Lawrence, 2016), the plots were based on the package ggplot2 (Wickham, 2016). The significance criterion was set to *p* < 0.05, but given the exploratory nature of the present study, we decided to report effects of *p* < 0.1 as marginally significant, as they may be insightful for future studies.

## 3 Results

### 3.1 Rhythmic body movements while listening to the speech stream

**Figure 1:**
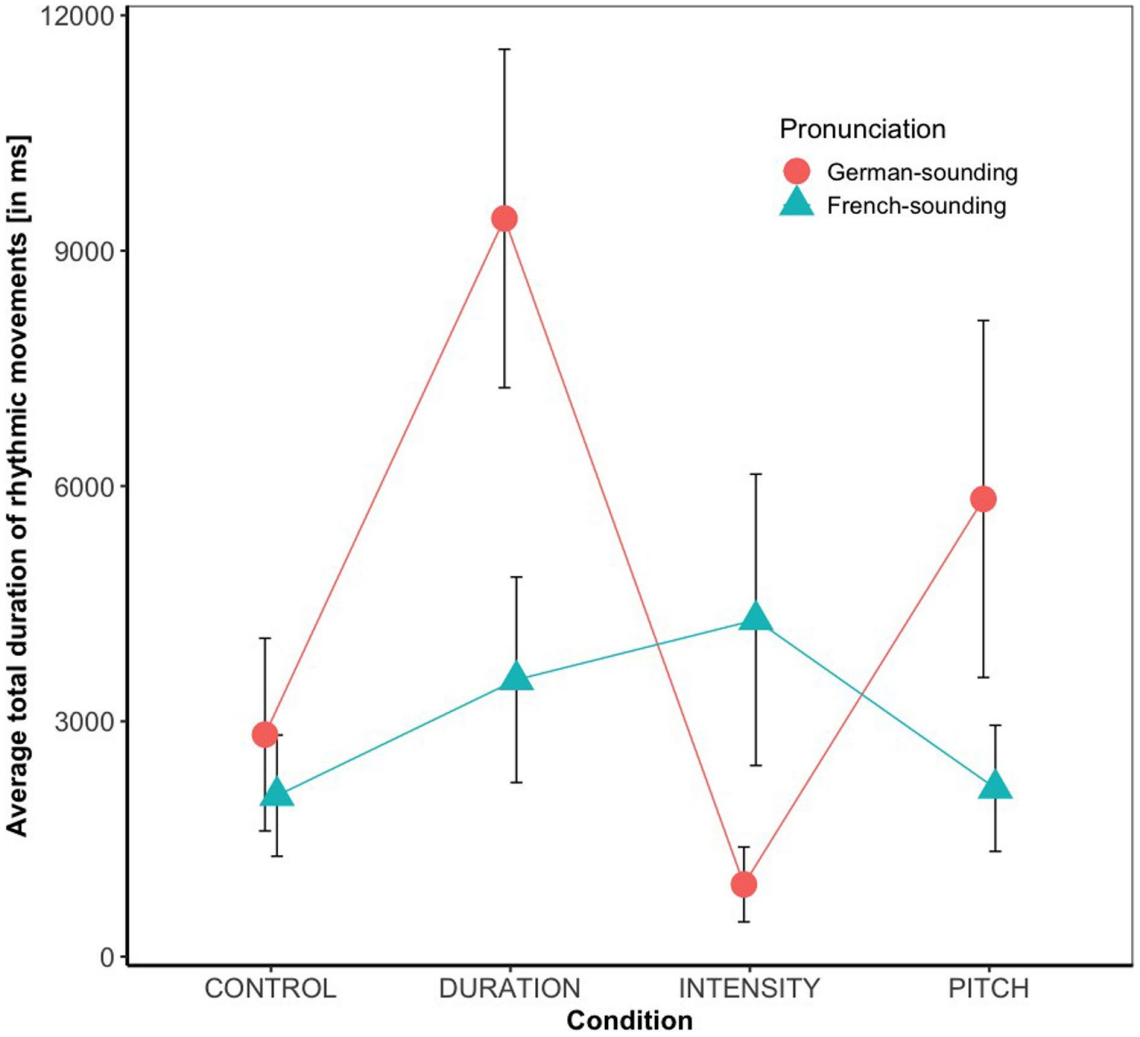
Average rhythmic body moving times (in ms) and their standard errors split by condition and pronunciation.

**Figure 2:**
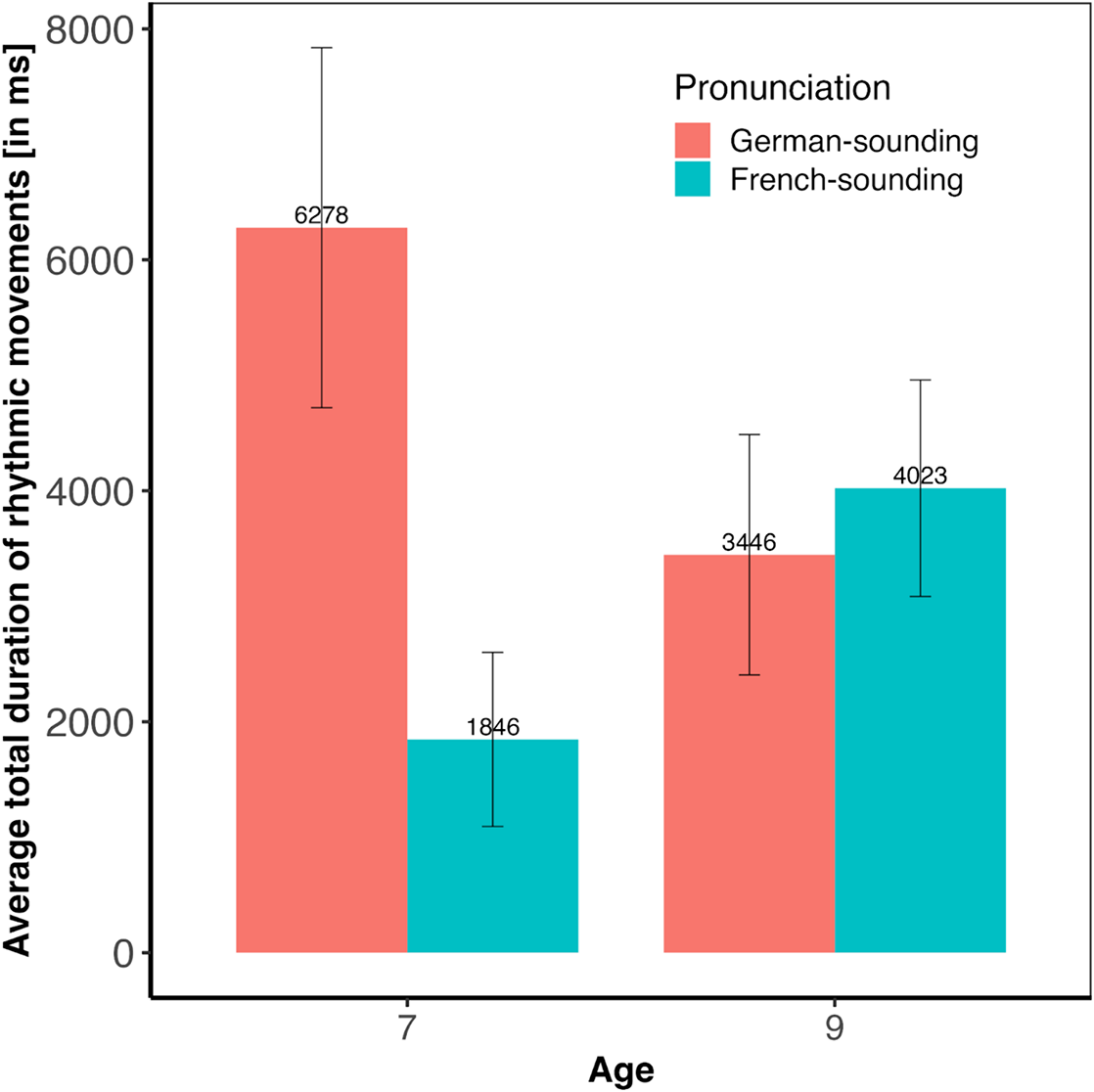
Average rhythmic body moving times (in ms) and their standard errors split by age and pronunciation.

Overall, 42% of the babies in our sample occasionally produced rhythmic movements while listening to speech stimuli. Results of the statistical analysis (see Figure 1) revealed a significant main effect of condition (*F*(3, 131) = 2.84, *p* < 0.05, with a small effect size of *η*^2^ = 0.06), and a significant Condition * Pronunciation interaction (*F*(3, 131) = 3.02, *p* < 0.05, with a moderate effect size of *η*^2^ = 0.07). There were no main effects of Age (*F*(1, 131) = 0.01, *p* = 0.92) or Pronunciation (*F*(1, 131) = 2.22, *p* = 0.14), no Condition * Age (*F*(3, 131) = 0.29, *p* = 0.83), and no Condition * Age * Pronunciation interaction (*F*(3, 131) = 0.68, *p* = 0.56). The Age * Pronunciation interaction (*F*(1, 131) = 2.91, *p* = 0.09) was marginally significant. Planned post-hoc tests revealed that the significant main effect of condition was due to significant differences between Duration versus Intensity (*p* < 0.05) and Duration versus Control (*p* < 0.05). An exploration of the Condition * Pronunciation interaction showed that the effect of condition was only significant with the German- (*F*(3, 65) = 4.43, *p* < 0.01) but not with the French-sounding pronunciation (*F*(3, 71) = 0.70, *p* = 0.55). Post-hoc pairwise comparisons within the subset of infants that had listened to the German pronunciation revealed significantly longer total moving times in the Duration compared to the Intensity (*p* < 0.01) and to the Control condition (*p* < 0.01), and marginally significantly longer moving times in the Pitch compared to the Intensity condition (*p* = 0.07). No other comparison reached significance. As the Age * Pronunciation did not reach significance, no further post-hoc comparisons were conducted, but Figure 2 suggests that the trend was that 7-month-olds moved more when listening to German- than when listening to French-sounding streams, while neither of the two pronunciations elicited more or less movements in 9-month-olds.

### 3.2 Correlations of rhythmic body movements and speech segmentation performance

**Figure 3:**
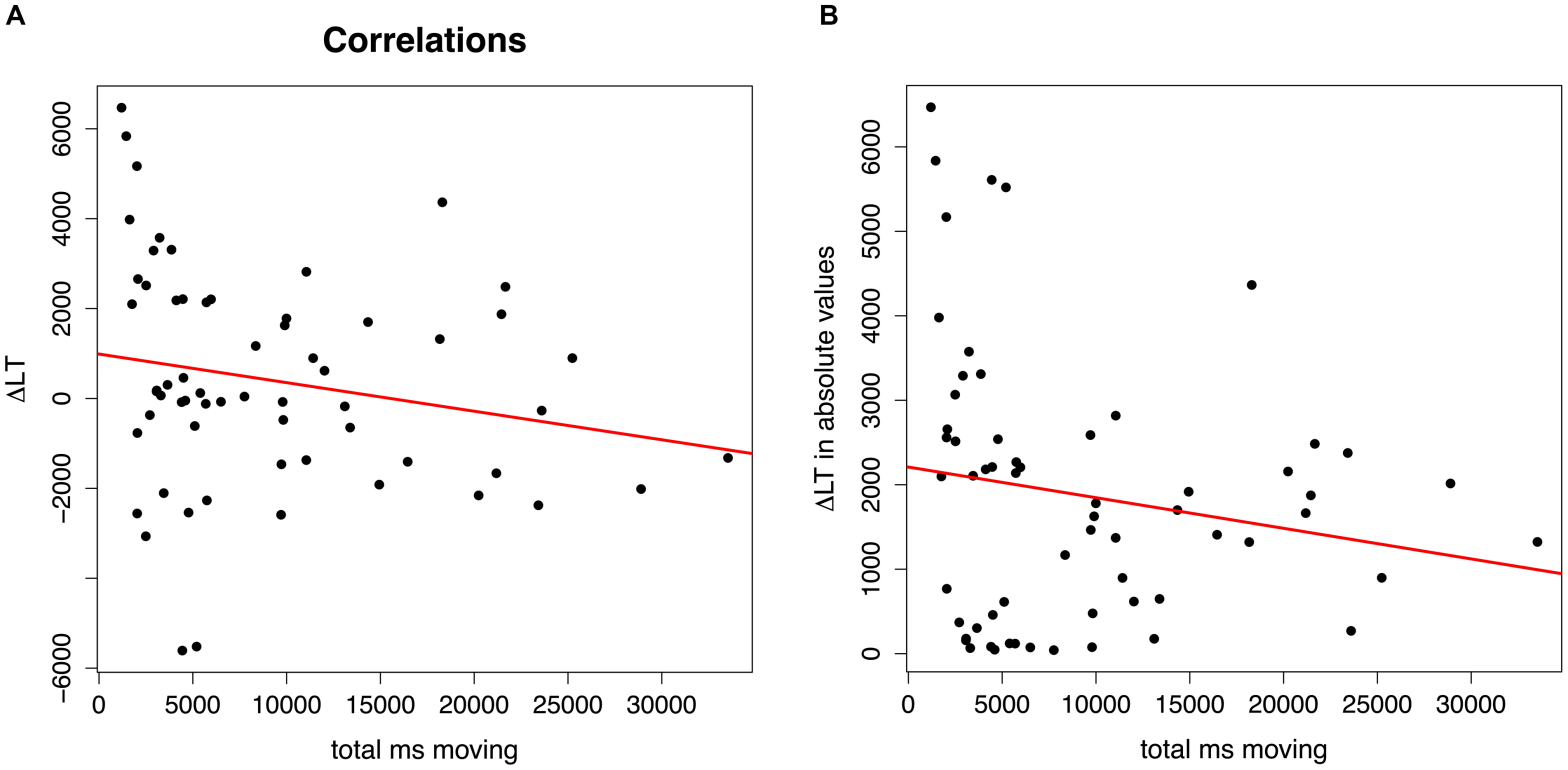
Plots of the correlations of infants’ total moving times (in ms) while listening to the artificial speech streams (x-axis) and their speech segmentation performance at test. In **(A)**, y-axis = Δ in looking time (Δ LT) between “strong-weak” or “weak-strong” trials in real numbers, with positive Δ LT scores reflecting longer looking times for test trials with syllable pairs that had occurred as weak-strong, and negative Δ LT scores for test trials with syllable pairs that had occurred as strong-weak during familiarization; in **(B)**, y-axis = Δ in looking time (Δ LT) in absolute values between “strong-weak” and “weak-strong” trials.

Both correlation analyses indicate an association between infants’ rhythmic movements while listening to the artificial language and their speech segmentation performance. The first correlation analysis revealed significantly longer rhythmic moving times with more negative looking time Δ scores (*r* = −0.26, *p* = 0.039), that is, when hearing “strong-weak” test trials (see Figure 3A). The second correlation analysis revealed a marginally significant correlation: the longer were the infants’ rhythmic moving times, the smaller were the absolute looking time Δ scores (*r* = −0.24, *p* = 0.059), that is, the weaker were their preferences for looking longer toward one of the two types of trials at test (see Figure 3B). Given the exploratory nature of the present study, and this *p*-value of 0.059 being very close to the significance criterion of *p* < 0.05, we will discuss this marginal effect as well as all significant effects below.

## 4 Discussion

The present study sought to explore infants’ rhythmic motor engagement while listening to rhythmic speech, asking whether infants’ rhythmic motor responses to rhythmic speech have a functional link with their language processing abilities. For this purpose, we analyzed 7.5- and 9.5-month-old German-learning infants’ rhythmic body movements to German- and French-sounding artificial language streams. We reasoned that rhythmic motor engagement may enhance infants’ perception of speech rhythm, which may be reflected in enhanced rhythmic motor responses to specific auditory rhythmic cues, namely pitch and duration. Moreover, we hypothesized that individual differences in infants’ early rhythmic engagement may be associated with their word segmentation abilities. Our analysis of infants’ reactions to the speech streams revealed that many infants moved rhythmically across conditions, but those who listened to duration-varied streams showed most rhythmic engagement. Moreover, the difference in rhythmic moving times between conditions was only present when infants listened to native, German-sounding language streams, but not when listening to non-native, French-sounding streams. Lastly, as expected, we found an association between infants’ rhythmic movements and their speech segmentation performance; but unexpectedly, those who moved less demonstrated more mature segmentation skills. We discuss these results in detail below.

The comparison of rhythmic moving times between the different acoustic conditions revealed that infants showed most rhythmic engagement when perceiving duration-based rhythm in speech. More specifically, their rhythmic moving times were longer in the duration than in both the intensity and control condition, and no statistical difference in their moving times was found when comparing the duration and pitch condition. This result was in line with our expectation, as it complements the original findings by Abboub et al. (2016), in which 7.5-month-olds did not show rhythm-based speech segmentation in the intensity and control condition, while they did in the duration condition. However, since Abboub et al. also found infants to show rhythm-based speech segmentation in the pitch condition, it was unexpected to find no statistical evidence for (or against) enhanced rhythmic movements in this condition, too. (However, as can be seen in Figure 1, there was a marginal effect of longer moving times in the pitch than in the intensity condition when stimuli were German-sounding, so it is possible that the lack of a significant effect is due to low power). In any case, the results suggest that infants’ body movements are indicative of their sensitivity to the auditory duration cues.

Notably, the above-described differences in rhythmic moving times between acoustic conditions were only attested by their reactions to speech synthesized from German but not French phonemes. This effect is in contrast with the behavioral results reported in Abboub et al. (2016) original study, where no effect of nativeness of pronunciation on infants’ speech segmentation was observed, which led the authors to suggest that language-specific perception may emerge later in development. In turn, the present findings indicate that German-learning infants are sensitive to native versus non-native pronunciation differences: seemingly, they recognize the non-nativeness of French-sounding speech, and find the native German-sounding speech more engaging as manifested in their rhythmic body response. This exploratory result can also be taken as evidence that the infants must have processed the synthesized artificial speech streams as speech-like. Since the presented stimuli were synthesized with a text-to-speech software (Dutoit et al., 1996) that drew on recordings of phonemes from a German (DE5) and a French (FR4) speaker, phoneme-level information was the only language-specific cue, as all prosodic information was later superimposed onto the synthesized stimuli. Hence, infants’ rhythmic motor engagement must have depended on their identification of the phoneme-level information as native-sounding.

Regarding the link between infants’ rhythmic motor responses to speech and their segmentation abilities, the correlational analyses revealed some interesting patterns. The first correlation analysis, which used infants’ Δ score in looking time in “strong-weak” versus “weak-strong” test trials attested that the longer infants had moved while listening to the language streams, the more they preferred listening to “strong-weak” trials at test, that is, to trials that followed from a trochaic grouping. It is possible that this result can be explained as a consequence of infants’ knowledge of the German prosodic system. Linguistically, German is described as a trochaic language (Wiese, 1996), and infants predominantly receive words with a strong-weak (trochaic) stress pattern in their input (Stärk et al., 2022). Experimental evidence suggests that already young German-learning infants have knowledge of their language’s predominant stress pattern, as indicated by their preferences to listen longer to trochaic than to iambic patterns in preferential looking paradigms between the ages of 4 and 6 months (Höhle et al., 2009, 2014; Marimon and Höhle, 2022). We may, hence, speculate that infants who produced more rhythmic movements to the rhythm of the speech streams found it more difficult to disengage from the perceived trochaic rhythm pattern and, hence, showed a trochaic listening preference at test, while infants with less motor engagement would more readily succeed at disengaging from the trochaic pattern and display instead a novelty preference for iambs at test. Since novelty preferences are generally interpreted to reflect more mature language processing skills (Hunter and Ames, 1988), this result may suggest that infants who showed less rhythmic engagement had more mature language segmentation abilities.

The second correlation analysis resulted in a marginally significant association between infants’ rhythmic moving times and the Δ score in looking time turned into absolute values. This result, which we need to interpret with caution as it did not fully reach our significance criterion, draws a link between infants’ rhythmic motor responses and the strength of a segmentation preference (without considering whether the preference was for trials reflecting a specific grouping, a familiarity or novelty preference). However, the direction of this association was unexpected: the longer infants had moved while listening to the language streams, the less strong was their preference. Like the results of the first correlation analysis, this second correlation also suggests that segmentation performance was lower when infants were more rhythmically engaged with the perceived rhythm. This raises the question of whether rhythmic motor engagement with speech rhythms has an inhibiting effect on language acquisition. Two explanations for the found association are possible: First, it may be that infants with more rhythmic movements were simply less attentive during the task. A second possibility is that the results reflect individual differences in whether the infants paid more attention to the rhythm of the auditory signal or to the specific syllable orders (i.e., phoneme-level information), with the former enhancing rhythmic motor responses and the latter enhancing segmentation performance. This is plausible, as studies with adults report that listeners can switch between focusing on musical or linguistic information when listening to music (e.g., Schön et al., 2005) or speech, and that whether one focuses more on the musical or linguistic properties is subject to individual variation (e.g., Rathcke et al., 2021). This interpretation is also in line with results of a recent study (Marimon et al., 2022) that probed whether German-learning 9-month-old infants rely on prosodic or syllable co-occurrence cues for segmenting an artificial language stream if the two cues are in conflict. Infants who relied more on prosody had stronger grammar skills at 3 years, whereas infants who relied more on syllable co-occurrences had larger vocabulary outcomes. This may suggest that, at least for infants between 7 and 9 months of age, focusing attention on rhythmic information rather than phoneme-level information may be a sign of a less mature response that is related to both a reduced ability to segment words from speech (the present finding) and a reduced ability to build up a large vocabulary (Marimon et al., 2022), which is interesting in light of proposals that vocabulary acquisition depends on speech segmentation (e.g., Jusczyk, 1997; Junge et al., 2012). Overall, the results suggest that infants’ general auditory preferences as well as differences in novelty and familiarity preferences typically observed in looking-time paradigms (e.g., Bergmann and Cristia, 2016; Gasparini et al., 2021) may not only be driven by stimulus familiarity or novelty, task complexity, or the infant age (Hunter and Ames, 1988), but may also result from bodily engagement with the rhythm of auditory stimuli. It would be ideal to replicate this study and to clarify whether infants who produce less rhythmic movements are indeed more mature in their language development, for example, by gathering information on their acquisition milestones (e.g., on their babbling, see Hoareau et al., 2019).

This finding of the individual differences raises the question of whether infants in our study focused either *only* on rhythm or only on phoneme-level information. However, this is highly unlikely: first, a strong preference for one test trial type over another at test (i.e., in infants that may have focused on phoneme-level information) can only occur if infants have perceived the rhythm cue that would bias them toward perceiving a grouping of syllables into pairs. Second, for the infants who moved more (i.e., infants that may have focused on rhythm), we found that their quantity of rhythmic movements was higher when they listened to native rather than non-native language streams, and since nativeness was related to the acoustic properties of phoneme-level information, it is not possible to conclude that these infants ignored one of the levels (phonemic or rhythmic) either. Hence, it is more likely that at the ages between 7 and 9 months, infants perceive both rhythm and phoneme-level information, with individual differences leading some infants to focus more on rhythm and others on syllable co-occurrence characteristics in the language streams.

The present study shows for the first time that infants learning the same language demonstrate individual differences in rhythmic grouping. Prior research by Yoshida and colleagues (Yoshida et al., 2010) had already established that infants’ rhythmic grouping is influenced by language experience: when exposed to streams of sounds alternating in duration, English-learning 7-8-month-olds favored a weak-strong (iambic) grouping, while Japanese-learning infants favored a strong-weak (trochaic) grouping at the same age. The authors related this cross-linguistic difference to infants’ experience with different word orders, with English showing many phrases with head-complement order (i.e., short function words before long content words), and Japanese showing many phrases with complement-head order (i.e., long content words before short function words). Differences within a group of individuals with the same native language had, so far, only been attested for German-speaking adults, who showed more consistent rhythmic grouping if they had higher musical rhythm perception acuity (Boll-Avetisyan et al., 2017, 2020a). Hence, the present research adds further evidence to the body of research indicating individual variability in infants’ speech perception and processing, which may systematically relate to internal or external factors beyond their language experience.

Our study is one of the first behavioral studies to tie early motor engagement with speech rhythm and language acquisition. Whereas spontaneous rhythmic movements in infants cannot be used as a reliable measure of tracking the auditory signal (Mandke and Rocha, 2023), our results suggest that they may still be indicative of the underlying speech perception processes. For example, while previous research has shown that infants’ passive sensorimotor experience (i.e., being moved to certain beats) reinforces their rhythm perception (Phillips-Silver and Trainor, 2005), our evidence contributes to it by demonstrating that infants’ active sensorimotor engagement may also play a role in rhythm processing. Moreover, according to our findings, rhythmic engagement in infancy may indeed modulate speech processing, e.g., in speech segmentation tasks. However, since the present results indicate reduced rather than enhanced speech processing in rhythmically moving infants, our findings add a new perspective to the body of research on infants’ improved sensitivity to auditory rhythms in multimodal contexts (Bahrick and Lickliter, 2000; Lewkowicz, 2000).

Previous accounts have suggested that infants’ enhanced rhythmic movements may be related to the positive affect that infants experience when listening to music (Zentner and Eerola, 2010), or that it could be a precursor of later entrainment abilities (Provasi et al., 2014; de l’Etoile et al., 2020), broadening the role of such engagement beyond mere manifestations of infants’ arousal. It has also been suggested that rhythmic entrainment may play a functional role in language acquisition. For example, educators have suggested that rhythmic movement with nursery rhymes and songs can help children to understand rhymes in language (e.g., Berger Cardany, 2013). The present results, however, do not provide support for this possibility. Instead, they suggest that for 7 to 9 month old infants motor engagement with auditory rhythms may actually hinder them from perceiving rhythmic grouping in spoken language – an important aspect in speech processing. We need to emphasize that the present results are correlational and based on exploratory analyses; hence this effect clearly requires replication before any conclusions can be drawn regarding the functional role of infants’ rhythmic body movements to auditory rhythms. Future studies will need to further explore the conditions that might enhance infants’ rhythmic movements, and how these associate with their language processing and language abilities. In order to get a better understanding of the causal link between rhythmic motor movements and rhythmic speech perception in language development, it might also be interesting to investigate the effects of hindering infants from moving their bodies on their speech processing (similar to Bruderer et al. (2015) and Yeung and Werker (2013), who showed that infants’ phoneme perception ability was affected, if they sucked on toys or pacifiers that constrained their lip movements).

A limitation of the present study is that it was not specifically designed to investigate infants’ body movements to speech rhythm. Consequently, the original design with numerous conditions and age groups was not ideal for the purpose. For example, we were not able to run correlation analyses that would probe the association of rhythmic motor responses and speech segmentation performance for each age group and each condition separately due to the limited observations after exclusion of the non-moving infants. Future studies should build on this exploration with study designs that are well-suited for establishing such correlations. Moreover, with the pre-existing data we had at hand, it was not possible to establish a baseline for probing whether infants who move more in silence also show more rhythmic movements while listening to speech. While not all prior studies used such baselines (Zentner and Eerola, 2010; Ilari, 2015; Nguyen et al., 2023), some indeed used them (Fujii et al., 2014; de l’Etoile et al., 2020), and future studies should consider collecting such data. Furthermore, the scope of this research could also be extended to other age groups in future studies. For example, it could be interesting to study whether rhythmic engagement with speech rhythm is more relevant for auditory processing in younger, pre-lexical age groups and whether rhythmic engagement with speech changes in maturation. Another direction for future research could be to examine whether and how rhythmic engagement to speech is modulated by infants’ experience with music and musical rhythms. Music experience in infancy has been previously shown to influence language acquisition (Langus et al., 2023; for a review, see Nayak et al., 2022), and music and speech rhythm have been suggested to share a number of properties aiding language processing (Fiveash et al., 2021). Lastly, it would be insightful to obtain more sensitive measures of infants’ rhythmic movements in the future by employing motion capture systems or even electromyography, to measure subtle muscle responses.

In sum, the present study provides the first exploratory evidence that infants’ spontaneous rhythmic body movements while listening to rhythmic speech are systematic. We observe that infants’ motor engagement is highest when they hear duration cues to rhythm. Moreover, they produce more rhythmic movements to native than to non-native speech. Lastly, individual differences in the quantity of infants’ rhythmic motor responses is associated with their speech segmentation ability, but other than expected, infants who showed less rhythmic movements showed more mature and stronger performance in speech segmentation. Future studies should further investigate how rhythmic body movements to speech rhythm may interact with language acquisition.

## Data availability statement

The pre-processed data supporting the conclusions of this article will be made available by the authors upon request.

## Ethics statement

The studies involving humans were approved by the ethics committee of the University of Potsdam. The studies were conducted in accordance with the local legislation and institutional requirements. Written informed consent for participation in this study was provided by the participants’ legal guardians/next of kin.

## Author contributions

NB-A: Writing – review & editing, Writing – original draft, Visualization, Validation, Supervision, Resources, Project administration, Methodology, Investigation, Formal analysis, Data curation, Conceptualization. AS: Writing – review & editing, Writing – original draft, Validation, Methodology. AL: Writing – review & editing, Conceptualization.

## References

[ref1] Abboub N. Boll-Avetisyan N. Bhatara A. Höhle B. Nazzi T. (2016). Rhythmic grouping according to the iambic-trochaic law in French- and German-learning infants. Front. Hum. Neurosci. 10:292. doi: 10.3389/fnhum.2016.00292 27378887 PMC4906042

[ref2] Aslin R. N. Saffran J. R. Newport E. L. (1998). Computation of conditional probability statistics by 8-month-old infants. Psychol. Sci. 9, 321–324. doi: 10.1111/1467-9280.00063

[ref3] Bahrick L. E. Lickliter R. (2000). Intersensory redundancy guides attentional selectivity and perceptual learning in infancy. Dev. Psychol. 36, 190–201. doi: 10.1037/0012-1649.36.2.190, PMID: 10749076 PMC2704001

[ref4] Berger Cardany A. (2013). Nursery rhymes in music and language literacy. Gen. Music Today 26, 30–36. doi: 10.1177/1048371312462869

[ref5] Bergmann C. Cristia A. (2016). Development of infants' segmentation of words from native speech: a meta-analytic approach. Dev. Sci. 19, 901–917. doi: 10.1111/desc.12341, PMID: 26353859

[ref6] Bhatara A. Boll-Avetisyan N. Agus T. Höhle B. Nazzi T. (2016). Language experience affects grouping of musical instrument sounds. Cogn. Sci. 40, 1816–1830. doi: 10.1111/cogs.12300, PMID: 26480958

[ref7] Bhatara A. Boll-Avetisyan N. Unger A. Nazzi T. Höhle B. (2013). Native language affects rhythmic grouping of speech. J. Acoust. Soc. Am. 134, 3828–3843. doi: 10.1121/1.4823848, PMID: 24180792

[ref8] Bion R. A. Benavides-Varela S. Nespor M. (2011). Acoustic markers of prominence influence infants’ and adults’ segmentation of speech sequences. Lang. Speech 54, 123–140. doi: 10.1177/0023830910388018, PMID: 21524015

[ref9] Boll-Avetisyan N. Bhatara A. Höhle B. (2017). Effects of musicality on the perception of rhythmic structure in speech. Lab. Phonol. 8:9. doi: 10.5334/labphon.91

[ref10] Boll-Avetisyan N. Bhatara A. Höhle B. (2020a). Processing of rhythm in speech and music in adult dyslexia. Brain Sci. 10:261. doi: 10.3390/brainsci10050261, PMID: 32365799 PMC7287596

[ref11] Boll-Avetisyan N. Bhatara A. Unger A. Nazzi T. Höhle B. (2016). Effects of experience with L2 and music on rhythmic grouping by French listeners. Biling. Lang. Congn. 19, 971–986. doi: 10.1017/S1366728915000425

[ref12] Boll-Avetisyan N. Bhatara A. Unger A. Nazzi T. Höhle B. (2020b). Rhythmic grouping biases in simultaneous bilinguals. Biling. Lang. Congn. 23, 1070–1081. doi: 10.1017/S1366728920000140

[ref13] Bolton T. L. (1894). Rhythm. Am. J. Psychol. 6, 145–238. doi: 10.2307/1410948

[ref14] Bril B. Sabatier C. (1986). The cultural context of motor development: postural manipulations in the daily life of Bambara babies (Mali). Int. J. Behav. Dev. 9, 439–453. doi: 10.1177/016502548600900403

[ref15] Brown S. Martinez M. J. Parsons L. M. (2004). Passive music listening spontaneously engages limbic and paralimbic systems. Neuroreport 15, 2033–2037. doi: 10.1097/00001756-200409150-00008, PMID: 15486477

[ref16] Bruderer A. G. Danielson D. K. Kandhadai P. Werker J. F. (2015). Sensorimotor influences on speech perception in infancy. Proc. Natl. Acad. Sci. 112, 13531–13536. doi: 10.1073/pnas.1508631112, PMID: 26460030 PMC4640749

[ref17] Christophe A. Dupoux E. Bertoncini J. Mehler J. (1994). Do infants perceive word boundaries? An empirical study of the bootstrapping of lexical acquisition. J. Acoust. Soc. Am. 95, 1570–1580. doi: 10.1121/1.408544, PMID: 8176060

[ref18] Condon W. S. Sander L. W. (1974). Neonate movement is synchronized with adult speech: interactional participation and language acquisition. Science 183, 99–101. doi: 10.1126/science.183.4120.99, PMID: 4808791

[ref19] Cristia A. Seidl A. Junge C. Soderstrom M. Hagoort P. (2014). Predicting individual variation in language from infant speech perception measures. Child Dev. 85, 1330–1345. doi: 10.1111/cdev.12193, PMID: 24320112

[ref20] Crowhurst M. J. Olivares A. T. (2014). Beyond the iambic-trochaic law: the joint influence of duration and intensity on the perception of rhythmic speech. Phonology 31, 51–94. doi: 10.1017/S0952675714000037

[ref21] Cummins F. (2009). Rhythm as entrainment: the case of synchronous speech. J. Phon. 37, 16–28. doi: 10.1016/j.wocn.2008.08.003

[ref22] de l’Etoile S. K. Bennett C. Zopluoglu C. (2020). Infant movement response to auditory rhythm. Percept. Mot. Skills 127, 651–670. doi: 10.1177/0031512520922642, PMID: 32389057

[ref23] De Ruiter J. P. (1998). Gesture and speech production. Nijmegen: Katholieke Universiteit.

[ref24] Dutoit T. Pagel V. Pierret N. Bataille F. Van der Vrecken O. (1996). The MBROLA project: towards a set of high quality speech synthesizers free of use for non commercial purposes. In Proceeding of fourth international conference on spoken language processing. Philadelphia, PA: IEEE.

[ref25] Echols C. H. Crowhurst M. J. Childers J. B. (1997). The perception of rhythmic units in speech by infants and adults. J. Mem. Lang. 36, 202–225. doi: 10.1006/jmla.1996.2483

[ref26] ELAN. (Version 4.9.4) [Computer software] (2016). Nijmegen: Max Planck Institute for Psycholinguistics, The Language Archive. Available at: https://archive.mpi.nl/tla/elan

[ref27] Esteve-Gibert N. Prieto P. (2014). Infants temporally coordinate gesture-speech combinations before they produce their first words. Speech Comm. 57, 301–316. doi: 10.1016/j.specom.2013.06.006

[ref28] Fiveash A. Bedoin N. Gordon R. L. Tillmann B. (2021). Processing rhythm in speech and music: shared mechanisms and implications for developmental speech and language disorders. Neuropsychology 35, 771–791. doi: 10.1037/neu0000766, PMID: 34435803 PMC8595576

[ref29] Friederici A. D. Friedrich M. Christophe A. (2007). Brain responses in 4-month-old infants are already language specific. Curr. Biol. 17, 1208–1211. doi: 10.1016/j.cub.2007.06.011, PMID: 17583508

[ref30] Fujii S. Watanabe H. Oohashi H. Hirashima M. Nozaki D. Taga G. (2014). Precursors of dancing and singing to music in three-to four-months-old infants. PLoS One 9:e97680. doi: 10.1371/journal.pone.0097680, PMID: 24837135 PMC4023986

[ref31] Gasparini L. Langus A. Tsuji S. Boll-Avetisyan N. (2021). Quantifying the role of rhythm in infants' language discrimination abilities: a meta-analysis. Cognition 213:104757. doi: 10.1016/j.cognition.2021.104757, PMID: 34045072

[ref32] Gleitman L. R. Wanner E. (1982). “Language acquisition: the state of the state of the art” in Language acquisition: The state of the art. eds. Wanner E. Gleitman L. R. (New York: Cambridge University Press), 3–48.

[ref33] Guellai B. Langus A. Nespor M. (2014). Prosody in the hands of the speaker. Front. Psychol. 5:700. doi: 10.3389/fpsyg.2014.00700 25071666 PMC4083345

[ref34] Hay J. S. Diehl R. L. (2007). Perception of rhythmic grouping: testing the iambic/trochaic law. Percept. Psychophys. 69, 113–122. doi: 10.3758/BF03194458, PMID: 17515221

[ref35] Hay J. F. Saffran J. R. (2012). Rhythmic grouping biases constrain infant statistical learning. Infancy 17, 610–641. doi: 10.1111/j.1532-7078.2011.00110.x, PMID: 23730217 PMC3667627

[ref36] Hayes B. (1995). Metrical stress theory: Principles and case studies. Chicago: University of Chicago Press.

[ref37] Hirsh-Pasek K. Nelson D. G. K. Jusczyk P. W. Cassidy K. W. Druss B. Kennedy L. (1987). Clauses are perceptual units for young infants. Cognition 26, 269–286. doi: 10.1016/S0010-0277(87)80002-1, PMID: 3677573

[ref38] Hoareau M. Yeung H. H. Nazzi T. (2019). Infants’ statistical word segmentation in an artificial language is linked to both parental speech input and reported production abilities. Dev. Sci. 22:e12803. doi: 10.1111/desc.12803, PMID: 30681753

[ref39] Höhle B. Bijeljac-Babic R. Herold B. Weissenborn J. Nazzi T. (2009). Language specific prosodic preferences during the first half year of life: evidence from German and French infants. Infant Behav. Dev. 32, 262–274. doi: 10.1016/j.infbeh.2009.03.004, PMID: 19427039

[ref40] Höhle B. Pauen S. Hesse V. Weissenborn J. (2014). Discrimination of rhythmic pattern at 4 months and language performance at 5 years: a longitudinal analysis of data from German-learning children. Lang. Learn. 64, 141–164. doi: 10.1111/lang.12075

[ref41] Hunter M. A. Ames E. W. (1988). A multifactor model of infant preferences for novel and familiar stimuli. Adv. Infancy Res. 5, 69–95.

[ref42] Ilari B. (2015). Rhythmic engagement with music in early childhood: a replication and extension. J. Res. Music. Educ. 62, 332–343. doi: 10.1177/0022429414555984

[ref43] Iversen J. R. Patel A. D. Ohgushi K. (2008). Perception of rhythmic grouping depends on auditory experience. J. Acoust. Soc. Am. 124, 2263–2271. doi: 10.1121/1.2973189, PMID: 19062864

[ref44] Iverson J. M. Thelen E. (1999). Hand, mouth and brain. The dynamic emergence of speech and gesture. J. Conscious. Stud. 6, 19–40.

[ref45] Johnson E. K. Tyler M. D. (2010). Testing the limits of statistical learning for word segmentation. Dev. Sci. 13, 339–345. doi: 10.1111/j.1467-7687.2009.00886.x, PMID: 20136930 PMC2819668

[ref46] Junge C. Kooijman V. Hagoort P. Cutler A. (2012). Rapid recognition at 10 months as a predictor of language development. Dev. Sci. 15, 463–473. doi: 10.1111/j.1467-7687.2012.1144.x, PMID: 22709396

[ref47] Jusczyk P. W. (1997). Finding and remembering words: some beginnings by English-learning infants. Curr. Dir. Psychol. Sci. 6, 170–174. doi: 10.1111/1467-8721.ep10772947

[ref48] Jusczyk P. W. Aslin R. N. (1995). Infants′ detection of the sound patterns of words in fluent speech. Cogn. Psychol. 29, 1–23. doi: 10.1006/cogp.1995.1010, PMID: 7641524

[ref49] Jusczyk P. W. Houston D. M. Newsome M. (1999). The beginnings of word-segmentation in English-learning infants. Cogn. Psychol. 39, 159–207. doi: 10.1006/cogp.1999.0716, PMID: 10631011

[ref50] Kemler Nelson D. G. Jusczyk P. W. Mandel D. R. Myers J. Turk A. Gerken L. (1995). The head-turn preference procedure for testing auditory perception. Infant Behav. Dev. 18, 111–116. doi: 10.1016/0163-6383(95)90012-8

[ref51] Kirschner S. Tomasello M. (2009). Joint drumming: social context facilitates synchronization in preschool children. J. Exp. Child Psychol. 102, 299–314. doi: 10.1016/j.jecp.2008.07.005, PMID: 18789454

[ref52] Kuhl P. K. Andruski J. E. Chistovich I. A. Chistovich L. A. Kozhevnikova E. V. Ryskina V. L.. (1997). Cross-language analysis of phonetic units in language addressed to infants. Science 277, 684–686. doi: 10.1126/science.277.5326.684, PMID: 9235890

[ref53] Langus A. Boll-Avetisyan N. van Ommen S. Nazzi T. (2023). Music and language in the crib: early cross-domain effects of experience on categorical perception of prominence in spoken language. Dev. Sci. 26:13383. doi: 10.1111/desc.13383 36869433

[ref54] Langus A. Mehler J. Nespor M. (2017). Rhythm in language acquisition. Neurosci. Biobehav. Rev. 81, 158–166. doi: 10.1016/j.neubiorev.2016.12.012 27993604

[ref55] Langus A. Seyed-Allaei S. Uysal E. Pirmoradian S. Marino C. Asaadi S.. (2016). Listening natively across perceptual domains. J. Exp. Psychol. Learn. Mem. Cogn. 42, 1127–1139. doi: 10.1037/xlm0000226, PMID: 26820498

[ref56] Lawrence M. A. (2016). Ez: easy analysis and visualization of factorial experiments. Available at: https://CRAN.R-project.org/package=ez

[ref57] Lewkowicz D. J. (2000). The development of intersensory temporal perception: an epigenetic systems/limitations view. Psychol. Bull. 126, 281–308. doi: 10.1037/0033-2909.126.2.281, PMID: 10748644

[ref58] Lew-Williams C. Saffran J. R. (2012). All words are not created equal: expectations about word length guide infant statistical learning. Cognition 122, 241–246. doi: 10.1016/j.cognition.2011.10.007 22088408 PMC3246061

[ref59] Mandke K. Rocha S. (2023). “Neural and behavioural rhythmic tracking during language acquisition: the story so far” in Rhythms of speech and language. eds. Meyer L. Strauss A. (Cambridge: Cambridge University Press).

[ref60] Marimon M. Höhle B. (2022). Testing prosodic development with the Headturn preference procedure: a test-retest reliability study. Infant Child Dev. 31:e2362. doi: 10.1002/icd.2362

[ref61] Marimon M. Höhle B. Langus A. (2022). Pupillary entrainment reveals individual differences in cue weighting in 9-month-old German-learning infants. Cognition 224:105054. doi: 10.1016/j.cognition.2022.105054, PMID: 35217262

[ref62] Masataka N. (1993). Effects of contingent and noncontingent maternal stimulation on the vocal behaviour of three-to four-month-old Japanese infants. J. Child Lang. 20, 303–312. doi: 10.1017/S0305000900008291, PMID: 8376471

[ref63] Mazokopaki K. Kugiumutzakis G. (2009). “Infant rhythms: expressions of musical companionship” in Communicative musicality: Exploring the basis of human companionship. eds. Malloch S. Trevarthen C. (Oxford: Oxford University Press), 185–208.

[ref64] McHugh M. L. (2012). Interrater reliability: the kappa statistic. Biochem. Med. 22, 276–282. doi: 10.11613/BM.2012.031, PMID: 23092060 PMC3900052

[ref65] Mersad K. Nazzi T. (2012). When mommy comes to the rescue of statistics: infants combine top-down and bottom-up cues to segment speech. Lang. Learn. Dev. 8, 303–315. doi: 10.1080/15475441.2011.609106

[ref66] Mintz T. H. Walker R. L. Welday A. Kidd C. (2018). Infants' sensitivity to vowel harmony and its role in segmenting speech. Cognition 171, 95–107. doi: 10.1016/j.cognition.2017.10.020, PMID: 29121588 PMC5818326

[ref67] Mundy-Castle A. (1980). “Perception and communication in infancy: a cross-cultural study” in The social foundations of language and thought: essays in honor of J.S. Bruner. ed. Olson D. (New York: Norton), 231–253.

[ref68] Nayak S. Coleman P. L. Ladányi E. Nitin R. Gustavson D. E. Fisher S. E.. (2022). The musical abilities, pleiotropy, language, and environment (MAPLE) framework for understanding musicality-language links across the lifespan. Neurobiol. Lang. 3, 615–664. doi: 10.1162/nol_a_00079, PMID: 36742012 PMC9893227

[ref69] Nazzi T. Bertoncini J. Mehler J. (1998). Language discrimination by newborns: toward an understanding of the role of rhythm. J. Exp. Psychol. Hum. Percept. Perform. 2, 756–766. doi: 10.1037//0096-1523.24.3.756 9627414

[ref70] Nazzi T. Kemler Nelson D. G. Jusczyk P. W. Jusczyk A. M. (2000). Six-month-olds' detection of clauses embedded in continuous speech: effects of prosodic well-formedness. Infancy 1, 123–147. doi: 10.1207/S15327078IN0101_11, PMID: 32680315

[ref71] Nespor M. Shukla M. van de Vijver R. Avesani C. Schraudolf H. Donati C. (2008). Different phrasal prominence realizations in VO and OV languages. Lingue Linguaggio 7, 139–168. doi: 10.1418/28093

[ref72] Newman R. Bernstein Ratner N. Jusczyk A. M. Jusczyk P. W. Dow K. A. (2006). Infants’ early ability to segment the conversational speech signal predicts later language development: a retrospective analysis. Dev. Psychol. 42, 643–655. doi: 10.1037/0012-1649.42.4.643, PMID: 16802897

[ref73] Nguyen T. Reisner S. Lueger A. Wass S. V. Hoehl S. Markova G. (2023). Sing to me, baby: infants show neural tracking and rhythmic movements to live and dynamic maternal singing. Dev. Cogn. Neurosci. 64:101313. doi: 10.1016/j.dcn.2023.101313, PMID: 37879243 PMC10618693

[ref74] Papoušek M. (1992). “Early ontogeny of vocal communication in parent–infant interactions” in Nonverbal vocal communication: comparative and developmental approaches. eds. Papoušek H. Jürgens U. Papoušek M. (Cambridge: Cambridge University Press), 230–261.

[ref75] Papoušek M. Papoušek H. Symmes D. (1991). The meanings of melodies in motherese in tone and stress languages. Infant Behav. Dev. 14, 415–440. doi: 10.1016/0163-6383(91)90031-M

[ref76] Phillips-Silver J. Trainor L. J. (2005). Feeling the beat: movement influences infant rhythm perception. Science 308:1430. doi: 10.1126/science.1110922, PMID: 15933193

[ref77] Provasi J. Anderson D. I. Barbu-Roth M. (2014). Rhythm perception, production, and synchronization during the perinatal period. Front. Psychol. 5:1048. doi: 10.3389/fpsyg.2014.01048 25278929 PMC4166894

[ref78] R Core Team (2021). R: a language and environment for statistical computing. Vienna, Austria: R Foundation for Statistical Computing.

[ref79] Rathcke T. Falk S. Dalla Bella S. (2021). Music to your ears: sentence sonority and listener background modulate the “speech-to-song illusion”. Music Percept. 38, 499–508. doi: 10.1525/mp.2021.38.5.499

[ref80] Saffran J. R. Newport E. L. Aslin R. N. (1996). Statistical learning by 8-month-old infants. Science 274, 1926–1928. doi: 10.1126/science.274.5294.1926 8943209

[ref81] Schmidt-Kassow M. Kotz S. A. (2008). Entrainment of syntactic processing? ERP-responses to predictable time intervals during syntactic reanalysis. Brain Res. 1226, 144–155. doi: 10.1016/j.brainres.2008.06.017, PMID: 18598675

[ref82] Schön D. Gordon R. L. Besson M. (2005). Musical and linguistic processing in song perception. Ann. N. Y. Acad. Sci. 1060, 71–81. doi: 10.1196/annals.1360.006 16597752

[ref83] Singh L. Steven Reznick J. Xuehua L. (2012). Infant word segmentation and childhood vocabulary development: a longitudinal analysis. Dev. Sci. 15, 482–495. doi: 10.1111/j.1467-7687.2012.01141.x, PMID: 22709398 PMC3383643

[ref84] Stärk K. Kidd E. Frost R. L. (2022). Word segmentation cues in German child-directed speech: a corpus analysis. Lang. Speech 65, 3–27. doi: 10.1177/0023830920979016, PMID: 33517856 PMC8886305

[ref85] Thelen E. (1979). Rhythmical stereotypies in normal human infants. Anim. Behav. 27, 699–715. doi: 10.1016/0003-3472(79)90006-X, PMID: 556122

[ref86] Thelen E. (1981). Kicking, rocking, and waving: contextual analysis of rhythmical stereotypies in normal human infants. Anim. Behav. 29, 3–11. doi: 10.1016/S0003-3472(81)80146-7, PMID: 7235314

[ref87] Thiessen E. D. Saffran J. R. (2003). When cues collide: use of stress and statistical cues to word boundaries by 7-to 9-month-old infants. Dev. Psychol. 39, 706–716. doi: 10.1037/0012-1649.39.4.706, PMID: 12859124

[ref88] Vanoncini M. Boll-Avetisyan N. Elsner B. Hoehl S. Kayhan E. (2022). The role of mother-infant emotional synchrony in speech processing in 9-month-old infants. Infant Behav. Dev. 69:101772. doi: 10.1016/j.infbeh.2022.101772, PMID: 36137465

[ref89] Vanoncini M. Hoehl S. Elsner B. Wallot S. Boll-Avetisyan N. Kayhan E. (2024). Mother-infant social gaze dynamics relate to infant brain activity and word segmentation. Dev. Cogn. Neurosci. 65:101331. doi: 10.1016/j.dcn.2023.101331, PMID: 38113766 PMC10770595

[ref90] Venetsanou F. Kambas A. (2010). Environmental factors affecting preschoolers’ motor development. Early Childhood Educ. J. 37, 319–327. doi: 10.1007/s10643-009-0350-z

[ref91] Victora M. D. Victora C. G. Barros F. C. (1990). Cross-cultural differences in developmental rates: a comparison between British and Brazilian children. Child Care Health Dev. 16, 151–164. doi: 10.1111/j.1365-2214.1990.tb00647.x, PMID: 2350868

[ref92] Weissenborn J. Höhle B. (2001). Approaches to bootstrapping: Phonological, lexical, syntactic and neurophysiological aspects of early language acquisition. Philadelphia, PA: John Benjamins.

[ref93] Wickham H. (2016). Ggplot2: elegant graphics for data analysis. New York: Springer International Publishing.

[ref94] Wiese R. (1996). The phonology of German. Oxford: Clarendon.

[ref95] Wilson M. Wilson T. P. (2005). An oscillator model of the timing of turn-taking. Psychon. Bull. Rev. 12, 957–968. doi: 10.3758/BF03206432, PMID: 16615316

[ref96] Woodrow H. (1909). A quantitative study of rhythm: the effect of variations in intensity, rate and duration. Beijing, China: Science Press.

[ref97] Woodrow H. (1911). The role of pitch in rhythm. Psychol. Rev. 18, 54–77. doi: 10.1037/h0075201

[ref98] Yeung H. H. Werker J. F. (2013). Lip movements affect infants’ audiovisual speech perception. Psychol. Sci. 24, 603–612. doi: 10.1177/0956797612458802, PMID: 23538910

[ref99] Yoshida K. A. Iversen J. R. Patel A. D. Mazuka R. Nito H. Gervain J.. (2010). The development of perceptual grouping biases in infancy: a Japanese-English cross-linguistic study. Cognition 115, 356–361. doi: 10.1016/j.cognition.2010.01.005, PMID: 20144456

[ref100] Zentner M. Eerola T. (2010). Rhythmic engagement with music in infancy. Proc. Natl. Acad. Sci. U.S.A. 107, 5768–5773. doi: 10.1073/pnas.1000121107 20231438 PMC2851927

